# Rapid adaptation to mammalian sociality via sexually selected traits

**DOI:** 10.1186/1471-2148-13-81

**Published:** 2013-04-11

**Authors:** Adam C Nelson, Kevin E Colson, Steve Harmon, Wayne K Potts

**Affiliations:** 1 Department of Biology, University of Utah, Salt Lake City, UT 84112, USA; 2 Current address: Molecular and Cellular Biology, Harvard University, Cambridge, MA 02138, USA; 3 Institute of Arctic Biology, University of Alaska Fairbanks, 902 N. Koyukuk Dr, PO Box 757000, Fairbanks, AK, 99775, USA; 4 Oklahoma State College of Osteopathic Medicine, 1111 W. 17th St, Tulsa, OK, 74107, USA

**Keywords:** Social selection, Sexual selection, Mate choice, Chemical communication, Tradeoffs

## Abstract

**Background:**

Laboratory studies show that the components of sexual selection (e.g., mate choice and intrasexual competition) can profoundly affect the development and fitness of offspring. Less is known, however, about the total effects of sexual selection on offspring in normal social conditions. Complex social networks, such as dominance hierarchies, regulate the opportunity for mating success, and are often missing from laboratory studies. Social selection is an extended view of sexual selection that incorporates competition during sexual and nonsexual interactions, and predicts complex evolutionary dynamics. Whether social selection improves or constrains offspring fitness is controversial.

**Results:**

To identify fitness consequences of social selection, wild-derived mice that had bred under laboratory conditions for eight generations were re-introduced to naturalistic competition in enclosures for three consecutive generations (promiscuous line). In parallel, a control lineage bred in cages under random mate assignment (monogamous line). A direct competition experiment using second-generation animals revealed that promiscuous line males had greater reproductive success than monogamous line males (particularly during extra-territorial matings), in spite of higher mortality and equivalent success in social dominance and sperm competition. There were no major female fitness effects (though promiscuous line females had fewer litters than monogamous line females). This result suggested that selection primarily acted upon a sexually attractive male phenotype in the promiscuous line, a hypothesis we confirmed in female odor and mating preference trials.

**Conclusions:**

We present novel evidence for the strength of sexual selection under normal social conditions, and show rapid male adaptation driven largely by sexual trait expression, with tradeoffs in survivorship and female fecundity. Re-introducing wild-derived mice to competition quickly uncovers sexually selected phenotypes otherwise lost in normal colony breeding.

## Background

The relative importance of the indirect effects of sexual selection on offspring fitness is the subject of ongoing debate [1-4]. By eliminating other forms of reproductive competition, laboratory rodent experiments have revealed that mate choice can strongly affect offspring fitness. For example, in a 10 minute assay, female mice mated to males they preferred to associate with in a two-way mate choice apparatus had offspring with greater viability than females mated to males they did not prefer to associate with [5]; similar results were found in a male association preference assay [6]. Intrasexual competition can also have independent effects on offspring fitness, and mice with high competitive ability often have offspring with higher fitness than their non-dominant counterparts [7]. Finally, post-copulatory selection during controlled polyandrous mating has been shown to improve offspring fitness relative to controlled monogamous mating [8,9]. These studies show that the components of sexual selection affect offspring fitness under certain conditions. Less understood, however, is whether these results are emblematic of the cumulative effects of sexual selection in normal social conditions [3].

For social animals, sexual selection takes place within a complex social network. The opportunity for sexual selection is largely determined by the structure of the social environment because relatively few socially dominant individuals are able to monopolize reproduction [10,11]. Thus, social selection, an extended view of sexual selection, refers to differential mating success due to any form of social competition, including competition over social rank, food, resources, space, parental care or kinship [12,13]. Indirect fitness effects of social selection could improve or constrain offspring fitness in competitive populations. For instance, the social environment can impose constraints on mating decisions such that individuals are forced to mate with partners they would otherwise not prefer, resulting in decrements to offspring viability [5,14]. Alternatively, competition within the social environment could improve the opportunity for sexual selection by providing maximal information regarding the potential fitness of offspring [15,16].

Laboratory based measures of selection in socially competitive environments are mixed. In the fruit fly *Drosophila melanogaster*, there is abundant evidence that larvae conceived in socially competitive environments have a fitness *disadvantage*[17-19], though the opposite effect has also been observed [15]. Studies on flour beetles [20] and amphipods [21] suggest that mating in socially competitive arenas produces sex-specific indirect effects, where fitness is enhanced in sons and depleted in daughters. Missing from these experiments, however, is a naturalistic social context [22]. In the wild, for instance, *D. melanogaster* males compete for a limited number of positions on the feeding site, which is where female choice is normally exercised [23,24]. More studies are needed to understand how parameters of offspring fitness affect the dynamics of social and sexual selection.

Three primary theories address the evolution of sexually selected traits [25]. First, the good genes model predicts that viability, usually in males, is signaled through the expression of condition-dependent traits and preference evolves because choosy females produce offspring with higher viability. Zahavi’s handicap principle [26] is a good genes model proposing that the expression of secondary sexual traits comes at the cost of survivorship, and only highly fit individuals are able to afford to display “cheat proof” handicap signals. Second, the sexy sons model predicts that female choice evolves to favor sexually attractive males who produce attractive sons, but otherwise make no contribution to offspring viability. Third, the chase-away model proposes that male display traits originate via exploitation of preexisting sensory bias in females, who are induced to mate in a suboptimal manner. Females can then evolve resistance by no longer preferring the trait, and males are in turn selected to evolve an even more extreme display trait. This latter model is a specific case of sexual conflict, which predicts that, due to the divergent reproductive interests of the sexes, promiscuity can lead to selection on traits that increase fitness in one sex at the expense of fitness in the opposite sex [27].

Sexual selection pressures can also influence offspring fitness through mechanisms not attributed to inherited alleles (i.e., transgenerational effects) [4,28]. Parents, particularly mothers, can alter investment in offspring development in response to social cues or environmental stressors such as resource availability, and in turn affect offspring social competitiveness [29,30]. Although parental effects can adaptively prime offspring for the current environment, conflicts between optimal strategies of parents and offspring (e.g., in resource allocation) can also incur costs on offspring [28]. How inheritance mechanisms collectively respond to social selection is not well understood, again highlighting the need for empirical measures of indirect effects of social and sexual selection.

Wild house mouse (*Mus musculus*) populations range from few to hundreds of individuals per acre, and transitions between these density extremes frequently occur within a few reproductive cycles [31,32]. Low-density populations lack social structure, while high-density commensal populations are organized around discrete territories consisting of a socially dominant male and several dominant females [33,34]. Social and sexual selection should be more intense in high-density environments because relatively few individuals monopolize reproduction. Females, who typically mate with the locally dominant male and/or dominant males of neighboring territories, are thought to drive mate choice in this species [35,36]. Males are not known to mate outside of their territorial boundaries or engage in mate guarding, but nevertheless do engage in adaptive mate choice [6].

We report here a breeding experiment to assess the indirect effects of social and sexual selection in mice. Motivated by the observation that, in isolation, mate preference [5,6], intrasexual competition [7], and polyandry [8,9] all confer fitness benefits to offspring, we aimed to identify whether the effects of competition for mating success within a social environment also improved offspring fitness, and if so, to what degree and through which components of fitness.

We bred a “promiscuous” line in socially competitive enclosures where mice compete for mates, nesting sites, and social dominance, and a “monogamous” line where social selection is eliminated by caged, random mate assignment [37]. Mice bred within their designated social treatment for three consecutive generations. During breeding, promiscuous line females were transferred to cages for parturition to minimize environmental effects on offspring fitness. In the first test, we directly competed second-generation monogamous line and promiscuous line mice in enclosures and found that the promiscuous line had a fitness advantage through increased male reproductive success, which was almost exclusively due to an advantage during extra-territorial mating (defined as instances where females mate with males other than the locally dominant male in her resident territory).

Results from direct competition suggested that the promiscuous line gained a fitness advantage through a sexually attractive male phenotype. To identify the role of female preference in this effect, we then used third-generation mice in a laboratory mate choice assay and found that females had both odor and mating preferences for promiscuous line males over monogamous line males. These results show that social selection in mice has rapid effects on male offspring and favors the expression of secondary sexual traits.

## Methods

### Mice

Mice used in this experiment carry a wild-caught, outbred genetic background on which five known MHC haplotypes from laboratory strains have been introgressed through selective breeding [38]. This genetically diverse strain with well-characterized MHC haplotypes allowed us to control for MHC-mediated mating behaviors during direct competition and mate choice experiments (below). This colony had been maintained under laboratory conditions and enforced monogamy for eight generations prior to the selection experiment described here.

### Promiscuous line and monogamous line breeding design

**Figure 1:**
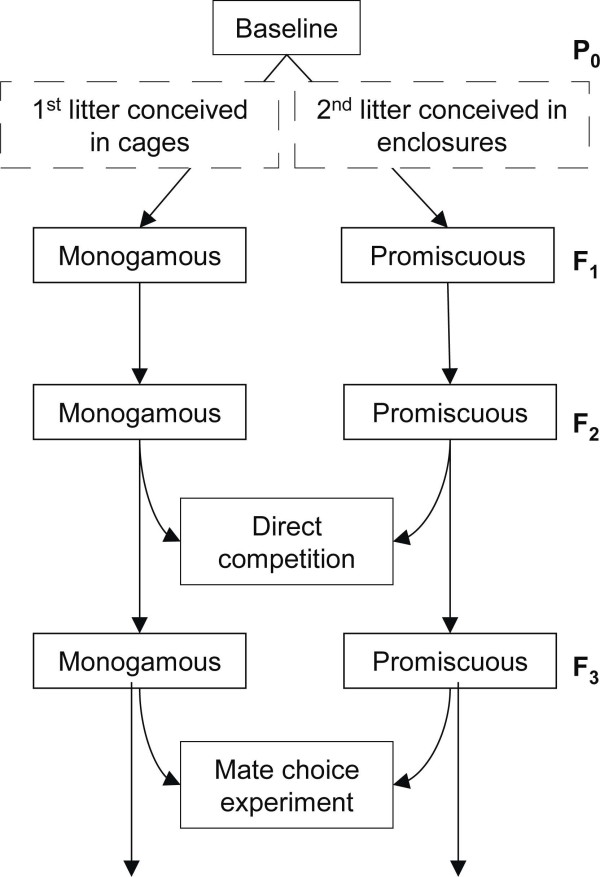
**Breeding design. **Monogamous boxes represent offspring conceived in random mate-assignment cages and promiscuous boxes represent offspring conceived in promiscuously breeding enclosures. Direct competition between second-generation, cage-born promiscuous line and monogamous line mice was conducted in semi-natural enclosures. Female odor and mating preferences for third-generation, cage-born promiscuous line and monogamous line mice was conducted in mate-choice arenas.

To determine the fitness effects of returning laboratory-adapted mice to social selection, mice were subjected to independent iterations of either continued enforced monogamy in cages or social selection in a seminatural environment where breeding is promiscuous (Figure 1). Natural selection for fecundity and viability were equalized between treatments by breeding the subsequent generation from an equal number of healthy offspring from an equal number of litters; both treatments were equally exposed to potential pathogens that may have been present in the animal facilities.

Initially, 50 P_0_ males and 50 females (age-matched and avoiding inbreeding) were bred monogamously to produce a single litter, which established the monogamous lineage. The same 100 adults were then randomly assigned to three independent seminatural population enclosures; pups from first litters conceived in the enclosures established the promiscuous lineage. Within each breeding treatment, random subsets of the resulting offspring were weaned at three weeks of age and used as the parental population for the subsequent generation. Both lineages were maintained at a potential effective population size (N_e_) of 100, but since some individuals do not breed due to social competition, the realized N_e_ of the promiscuous line was below 100.

We chose to initiate the monogamous line with the P_0_ first litter and the promiscuous line with the P_0_ second litter (as opposed to randomizing treatments between first and second litters) because it afforded a design with the most variables controlled: when collected from enclosures, females will vary in whether they have mated; some will have been dominant and others subordinate, and as a result differed in their access to resources; the time to first litter is longer and more variable in enclosures, introducing additional variation. Although we cannot exclude the possibility that starting the monogamous line first contributed to our results two and three generations later, previous work in our laboratory has shown that there are no fitness differences between first and second litters [39]. Consistently, we found no differences between the size of first and second litters as measured by the number of pups that survived to weaning age (means ± s.e.m.: first = 6.81 ± 0.376; second = 7.17 ± 0.381).

Promiscuous line enclosure populations consisted of 10 males and 20 females (on average) to reflect the female biased adult sex ratio commonly found in commensal populations [34]. To minimize differential effects of experience and environment on offspring from the promiscuous and monogamous lines, pregnant females from the promiscuous line were transferred from enclosures to cages for parturition. Because nongenetic effects may be a crucial mechanism for rapid adaptation to changes in the social environment, this design provides opportunity for transgenerational inheritance [40,41] by directly exposing parents to the two different social conditions. Experimental comparisons between treatments (i.e., direct competition and mate choice experiments, below) were performed on cage-born offspring from both lines; therefore, at the time of testing, promiscuous offspring had never experienced seminatural conditions, only their parents and ancestors had.

### Seminatural enclosures

The promiscuous lineage bred in seminatural enclosures that mimic the social ecology of commensal *Mus domesticus* in nature; the incidence of multiple paternity [42], population density [32], and sex ratio [33] are all consistent with measurements from natural populations. Each enclosure (ca. 22.2 m^2^) was divided into six subsections by climbable hardware cloth (45.72 cm) to create territorial boundaries. Four subsections were designated as “optimal territories” and had defendable nest boxes made of covered, opaque plastic bins (75.7 liters) with 5.08 cm diameter entryways and containing nesting materials and food. The remaining two subsections were designated as “suboptimal territories” that had light-exposed nest boxes (made of 61 cm × 20 cm planter boxes fitted with wire lids and 5.08 cm entryways) and adjacent open-access food containers. Together, optimal and suboptimal territorial boundaries created environmental complexity in which mice established social dominance hierarchies. Water was provided ad libitum in one-gallon poultry waterers. These enclosures have previously been found to elicit normal behavior in wild mice [36,39,43,44].

To prevent incidental breeding before the establishment of male social territories (the primary social unit in commensal mice), placeholder females were used at the onset of each population for one week before promiscuous line breeding females replaced them in the enclosures.

### Direct competition between second-generation promiscuous line and monogamous line

To determine whether animals from the promiscuous line gained a fitness advantage following two generations of sexual and social selection, we competed cage-born promiscuous line and monogamous line (n = 300) mice in 10 independent enclosures for 35 weeks (Figure 1). Each competition enclosure consisted of 10 male and 20 female founders; half were from the monogamous line, and half were from the promiscuous line. The influence of inbreeding was controlled by limiting the presence of relatives within an enclosure to one or a few pairs of female cousins, which were always equally balanced between treatments. We measured four fitness components among the founders: 1) male and female reproductive success using Y-chromosome and mitochondrial genetic markers; 2) individual reproductive success using parentage analysis; 3) social dominance and competitive ability using RFID tags; and 4) survivorship.

#### Male and female reproductive success

To test for sex-specific effects of social selection on fitness, the reproductive success of male and female founders of each treatment during direct competition was determined by tracking the inheritance of neutral, polymorphic Y-chromosome and mitochondrial genetic markers as described previously [39]. Approximately every 35 days we preformed “sweeps” through the enclosures to remove and sacrifice pups for genetic analysis (thus preventing them from reaching sexual maturity). Within each competition enclosure, males and females from the promiscuous line shared alleles on the Y-chromosome and mitochondrial genome that were different from the alleles shared by males and females from the monogamous line. Accordingly, treatment-level reproductive success for male founders was determined by genotyping male offspring, and female reproductive success was determined by genotyping all offspring.

To determine the number and size of litters produced by promiscuous line and monogamous line founders (of both sexes) during competition, litters were classified by their estimated age and size and their precise location. “Regular” litters had a single mitochondrial genotype, were found in one location and were of uniform age. “Mixed” litters had at least two mitochondrial genotypes, were found in multiple locations, had different age classes, or any combination thereof. “Communal” litters had more than 12 offspring of the same age. Litters were also classified by the presence of one or two Y chromosome markers, which identified a subset of litters sired by multiple males.

#### Individual reproductive success by parentage analysis

To determine the reproductive success of monogamous line and promiscuous line founders with known social dominance identities (below), we used paternity analysis to genotype parents and offspring from two competition enclosures using 10 unlinked autosomal microsatellite loci, as described previously [45]. Parentage was estimated using Cervus likelihood analysis software [46]. All parent offspring trios were found to have greater than 80% confidence. Identification of “mixed” (i.e., multiple paternity) litters was used to determine the effect of breeding treatment on reproductive success during postcopulatory sexual selection (sperm competition and cryptic female choice). We also analyzed the effects of breeding treatment on the size and number of “pure” (i.e., sired by a single male) litters.

#### Social dominance and territory defense

To determine social dominance and territorial abilities of the monogamous line and promiscuous line, founders were marked with unique passive integrated transponder (PIT) tags surgically implanted subdermally between the scapulas. Transceivers and readers (BioMark, Boise, ID) were placed at each of the optimal and suboptimal feeders in two competition enclosures at a time (hereafter “reader session”), and data streamed to a computer with data-logging software (Minimon, Culver City, CA). Transceivers were regularly rotated through the 10 enclosures throughout the competition experiment. Male social dominance was assigned when a male had > 90% of the PIT-tag reads at a single reader. At “undefended” locations no single male had 90% of the reads. Females move between territories more than males, and though they do not compete for exclusive representation at a single location, they do compete for positions at optimal territories as indicated by female-female agonistic interactions and higher reproduction in optimal territories (below). Thus, female social dominance was assigned when > 50% of a given female’s reads were at a single location.

PIT-tag data were used to determine treatment effects on social dominance ability in three ways. First, we compared the number of socially dominant males and females between treatments. Second, we used treatment-level (Y chromosome and mitochondrial genotyping) reproductive success measures to determine differences between promiscuous line and monogamous line mice that achieved social dominance (that is, the interaction between social dominance and reproductive success). Third, because treatment-level analysis showed that promiscuous line males sired a significant portion of offspring in territories defended by monogamous line males, we used parentage analysis to track the reproductive success and social dominance of individual males. In particular, we quantified the number of offspring born within the territory of their socially dominant father (“within-territory”), outside the territory of their socially dominant father (“extra-territory”), or in territories undefended by a dominant male (“undefended”).

#### Survivorship

Survivorship of founders (n = 300) was determined by periodic checks (approximately every 10 days) in each enclosure. Dead founders were identified by their PIT-tag.

#### Major histocompatibility complex

The major histocompatibility complex (MHC) is a substantial mediator of vertebrate fitness effects [47]. When assigning monogamous line and promiscuous line mice to competition enclosures, we used genetic markers of the five MHC haplotypes in this population to balance MHC allelic diversity between each treatment. As a result, MHC haplotype frequencies were equivalent between treatments at the onset of competition. By controlling for the opportunity of MHC-mediated selection, this strategy allowed us to better focus on the effects of social selection for two reasons. First, MHC-mediated mate choice (either due to heterozygote advantage, rare-allele advantage, or inbreeding avoidance) is well documented to have a profound fitness effects in both socially competitive and noncompetitive environments [48]. Second, MHC-mediated selection resulting from pathogen pressures could have introduced differential disease susceptibility profiles between the monogamous and promiscuous lines during the three generations of breeding [49]. Thus, by balancing MHC genotypes between treatments, we ensured that MHC-mediated selection would not obscure the effects of social selection.

### Female mate choice for third-generation promiscuous line vs. monogamous line males

To determine if the promiscuous line male fitness advantage during direct competition was driven by female odor and mating preferences, we used a three-cage mate choice arena and the Timescience (Salt Lake City, UT) recording system. The mate-choice arena consisted of two “male” cages connected by PVC tunnels to a single “female” cage (cage dimensions: 46 × 30 × 15 cm). An infrared, black and white camera was mounted above each arena. Each odor/mate preference trial consisted of two males and one female. Females were from both the promiscuous line (n = 6) and the monogamous line (n = 5). Males were given plastic collars, made of two small connected zip-ties whose protruding ends prevented them from leaving their designated cage. Prior to the experiment, males and females were housed individually in maintenance cages for one week and males were habituated to their collar for one day. Promiscuous line and monogamous line males were placed in alternating sides of the arena between trials to prevent bias. Males were age and weight matched, were unrelated to the female and to each other. Consistent with the direct competition experiment, we balanced the effects of MHC-mediated mate choice by ensuring that MHC haplotypes were equally represented in promiscuous line and monogamous line males.

This assay proceeded in three phases using F_3_ cage-born males from the promiscuous and monogamous lines as subjects. First, to assess male scent-marking behavior, we introduced one collared male from each treatment to each of two “male” cages of the arena. The males were allowed to scent mark the filter paper substrate of their cages for 30 minutes in the presence of a stimulus (soiled bedding from the test female’s sibling cage presented in an aerated canister). The area covered and number of scent marks were quantified with a Typhoon scanner (GE Healthcare Life Sciences, Piscataway, NJ) using the 532 nm ROX filter.

Second, to determine odor preferences, females were allowed to investigate the scent marks with the males removed from the arena (females did not wear collars). Time spent investigating scent marks was quantified. Third, to determine mating preferences, collared males were reintroduced to their cages in the arena; food, water and bedding were added to each cage. After four days, females were removed from the arena and the paternity of offspring from pregnant females was determined by genetic analysis as described. Due to a slow recording rate (frame per second) over the four-day experiment, intromissions could not be discriminated from ejaculations. The proportion of offspring sired by either male determined mating preference. All data were collected by participants blind to the treatment of the subjects.

### Weight analysis of third generation promiscuous line and monogamous line mice

To test for effects of breeding treatment on male and female weight, we recorded the mass of third-generation offspring 10 days after birth and then at 10-day intervals until the offspring were 60 days old. An average of 10 weighted individuals from 10 litters per treatment is reported.

### Statistics

We used general linear models (GLM) and general linear mixed models (GLMM) to determine the effects of social selection on offspring fitness and phenotype. Where appropriate, we used post hoc Tukey’s HSD tests to further evaluate significant parameters. Analyses were carried out with the JMP 9.0 statistical package (SAS Institute).

#### Direct competition: reproductive success

The effects of breeding history on reproductive success during direct competition were analyzed with GLMM using a restricted maximum likelihood approach. Treatment-level reproductive success measures (number of offspring, number of litters and litter size) were modeled as dependent variables for males (Y chromosome markers) and females (mitochondrial markers) separately. For all models, competition enclosure was modeled as a random factor, and offspring sweep and parental treatment as fixed factors. Because socially dominant, promiscuous line males had higher mortality than dominant monogamous line males, we also analyzed male reproductive success with number of surviving males (per treatment) as an additional factor. The interaction between male and female reproductive success was analyzed in a separate model by adding sex, treatment and their interaction a fixed effects.

Using parentage analysis, we used GLMM to detect treatment effects on the number of pups per litter (for female founders only), and the number of pups per pure and mixed litters (for male and female founders). Here, number of offspring per litter was the dependent variable and maternal treatment, paternal treatment, maternal by paternal interaction, and offspring sweep were added as fixed effects.

#### Direct competition: social dominance and survivorship

We used GLMM to determine treatment effects on social dominance on during direct competition. The number of dominant males and females were separately modeled as a dependent variable, with enclosure as a random factor, and PIT-tag reader session and breeding treatment as fixed factors. To analyze population-level and individual-level effects of male social dominance on reproductive success, the mean number of male offspring per territory type was fitted as a dependent variable, enclosure as a random variable, and PIT-tag reader session, paternal treatment, territory type (within territory, between territory and undefended) and their interaction were modeled as fixed factors. Survivorship was analyzed with the Cox-Mantel log-rank test.

#### Female mate choice experiment

Male scent marking behavior was analyzed using general linear models (GLM). Female odor and mating preferences were determined by measuring the proportion of time spent in the two male cages (promiscuous line male vs. monogamous line), and the proportion of offspring sired by either male. Proportions were normalized by arcsine-root transformation, and comparisons were made using paired t-tests.

#### Weight analysis

Weight data were analyzed using GLM with weight as the dependent variable and treatment, age, and their interaction as fixed factors.

## Results

### Direct competition: promiscuous line males have greater reproductive success (primarily through extra-territorial mating), and lower survivorship, compared to monogamous line males

#### Male and female reproductive success

**Figure 2:**
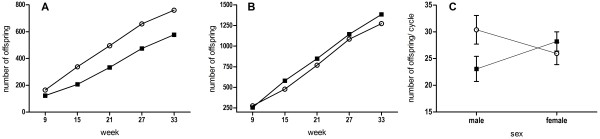
**Sex-specific reproductive success during direct competition. **Cumulative lifetime reproductive success (as measured by number of offspring over 35 weeks of competition) for promiscuous line (open circles) and monogamous line (filled squares) males (**A**) and females (**B**). Promiscuous line males had significantly more offspring than monogamous line males (Table 1). Male reproductive success values are calculated from number of sons only (see Methods). (**C**) interaction of sex-specific effects of breeding treatment: promiscuous line (circles) male reproductive success was negatively correlated with promiscuous line female reproductive success (P = 0.027). Shown are means and standard errors per offspring sweep cycle; male reproductive success values were multiplied by two.

We collected 2,738 pups from 466 litters, and used 2,655 mitochondrial genotypes and 1,337 Y chromosome genotypes from 10 independent competition enclosures to determine sex-specific reproductive success. Promiscuous line males sired 57% of the offspring born during competition, and monogamous line males sired 43%. Results from GLMM analysis (results summarized in Table 1) showed that promiscuous line males produced significantly more offspring (Figure 2A) and a greater number of litters, but did not have larger litters. Because promiscuous line males had a higher mortality rate (see below), they also had greater reproductive success per surviving male than monogamous line males.

**Table 1:** Male and female reproductive success during direct competition

**Response Variable**	**Source**	**Males**				**Females**			
		**DF**	**DFden**	***F***	***P***	**DF**	**DFden**	***F***	***P***
Number of offspring									
	Sweep^*^	4	84.5	0.94	0.45	4	82.7	0.75	0.56
	Breeding treatment	1	84.4	4.38	**0.039**	1	82.1	0.70	0.41
	Breeding treatment *x *number of surviving males	1	41.8	4.11	**0.049**				
Number of litters									
	Sweep	4	83.1	1.02	0.40	4	85	0.55	0.70
	Breeding treatment	1	82.6	5.82	**0.018**	1	85	6.59	**0.012**
Litter size									
	Sweep	4	72.8	1.22	0.31	4		83.8	0.27
	Breeding treatment	1	75.8	0.25	0.62	1		83.3	0.28

GLMM analysis of components of promiscuous line and monogamous line founder (n = 100) reproductive success as determined by sex-specific markers with enclosure (n = 10) as a random effect and all other variables as fixed effects.

* Offspring sweeps were conducted approximately every 35 days to remove and sacrifice pups for genetic analysis (thus preventing them from reaching sexual maturity) (n = 5).

Promiscuous line and monogamous line females had 48% and 52% of the offspring, respectively; these differences were not significant under GLMM analysis (Table 1; Figure 2B). Females from the promiscuous line had significantly fewer litters, but also had slightly (but not significantly) larger litters (Table 1).

A separate GLMM analysis with sex, treatment and their interaction as fixed factors found a significant interaction effect (Figure 2C) (GLMM: n = 198; treatment x sex *F* = 4.98, *P* = 0.027; all other factors *P* > 0.24), suggesting a negative correlation between male and female reproductive success in the promiscuous line.

#### Social dominance and territory defense

There were no treatment effects on the ability of males to acquire and defend territories (GLMM: n = 52; treatment *F* = 0.26, *P* = 0.62), nor were there differences in the number of socially dominant females (n = 52; treatment *F* = 0.018, *P* = 0.90).

**Figure 3:**
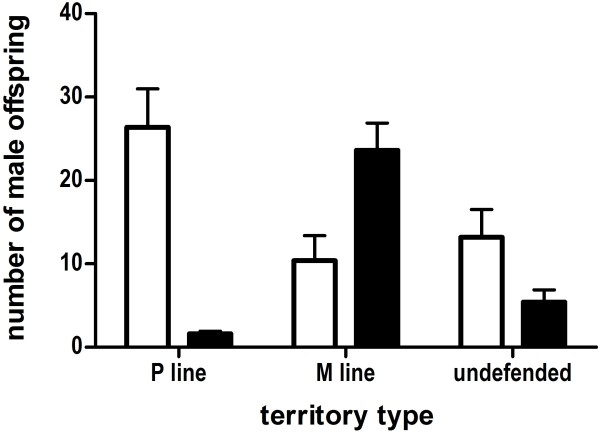
**Effects of breeding treatment on male social dominance and reproductive success.** Means and standard errors are calculated Y-chromosome markers from 10 competition enclosures. Promiscuous line males (open bars) had significantly more offspring than monogamous line males (closed bars) due to greater relative representation within territories defended by either promiscuous (P) line or monogamous (M) line males, and in undefended territories. In territories defended by promiscuous line males, 95% of offspring had promiscuous line paternity; in territories defended by monogamous line males, 68% of offspring had monogamous line paternity.

The majority of offspring (477/620, 77%) were born in defended territories (GLMM: *n* = 52; defended *F* = 46.7, *P* = <0.0001). To determine the relationship between social dominance and treatment-level measures of reproductive success, GLMM analysis of offspring counts with paternal treatment, territory type (defended by males from the promiscuous line or monogamous line, or undefended) and their interaction as factors found all three terms to be significant (treatment *F* = 8.23, *P* = 0.006; territory type *F* = 3.95, *P* = 0.026; interaction *F* = 23.78, *P* < 0.0001; Figure 3). Ninety-five percent of the offspring born in territories defended by a promiscuous line male had promiscuous line paternity; in contrast, just 68% of the offspring born in territories defended by a monogamous line male had monogamous line paternity. Promiscuous line males also had greater representation in undefended territories, with 61% of the offspring. Post-hoc Tukey’s HSD tests showed no additional significant effects. Thus, promiscuous line males had significantly greater fitness due to greater reproductive success in all three territory types.

#### Individual reproductive success by parentage analysis

**Figure 4:**
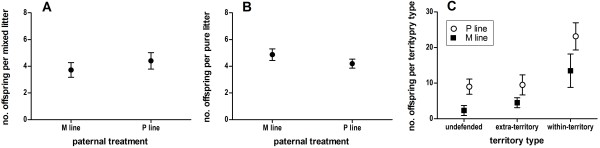
**Individual-level reproductive success of males during sperm competition and extra-territorial mating. **Mean and standard errors are calculated from two competition enclosures using parentage analysis. There were no effects of paternal breeding treatment on the size of mixed (multiple paternity) (**A**) or pure (single paternity) (**B**) litters. Individual promiscuous (P) line males (circles) had more offspring within their own defended territories (within-territory), inside the territories of neighboring dominant males (extra-territory), and in undefended territories than monogamous (M) line males (squares) (**C**).

To measure individual-level reproductive success, we genotyped promiscuous line and monogamous line founders (n = 60) and offspring (n = 559, comprising 110 litters) from two competition enclosures. Consistent with the treatment-level measurements, 57% of parent-typed offspring had promiscuous line paternity and 47% had promiscuous line maternity. First, we characterized all litters, irrespective of parental treatment, and found that 73% of all litters were of single paternity and 27% were of mixed paternity; mixed litters were significantly larger than pure litters (Means: mixed = 6.8 pure = 4.9; GLMM: *n* = 100; *F* = 10.3, *P* = 0.0019). We then used GLMM to analyze the effects of breeding treatment on the frequency and size of pure and mixed litters (Table 2). There were no paternal effects on the frequency or size of mixed or pure litters (Figures 4A,B), nor were there effects of maternal treatment or paternal *x* maternal interactions on mixed and pure litter size (Table 2).

**Table 2:** Results from parentage analysis of offspring born during direct competition

**Response variable**	**Source**	**DF**	**DFden**	***F***	***P***
Litter size					
	Sweep^*^	4	92.4	0.64	0.64
	Maternal treatment	1	93	0.63	0.43
	Litter type (mixed or pure)	1	92	10.3	**0.0019**
	Maternal treatment × litter type	1	92.0	0.93	0.34
No. pups per mixed litter					
	Sweep	4	12.7	0.36	0.83
	Maternal treatment	1	51.5	0.33	0.57
	Paternal treatment	1	51	0.87	0.35
	Maternal × paternal treatment	1	51	1.61	0.21
No. pups per pure litter					
	Sweep	4	67.8	0.79	0.54
	Maternal treatment	1	70.1	0.16	0.69
	Paternal treatment	1	65.0	1.34	0.25
	Maternal × paternal treatment	1	54.3	0.95	0.34
Number of offspring by territory type					
	PIT-tag reader session^#^	2	27	0.76	0.48
	Territory type^+^	2	27	10.4	**0.0005**
	Paternal treatment	1	27	8.13	**0.0083**
	Territory type × paternal treatment	2	27	0.30	0.74

Shown are the effects of parental treatment (monogamy or promiscuous) on the size of all litters, mixed (multiple paternity) litters, and pure (single paternity) litters. Also shown are the effects of paternal treatment and social dominance on number of offspring. GLMM analysis of reproductive success with population as a random effect and all else as fixed effects.

^+^Territory type: Within-territory, extra-territory or undefended.

^*^Offspring sweeps were conducted approximately every 35 days to remove and sacrifice pups for genetic analysis (thus preventing them from reaching sexual maturity) (n = 5).

^#^Transceivers were regularly rotated through the 10 enclosures throughout the competition experiment.

We then analyzed individual male reproductive success (from both treatments) by comparing the number of offspring born in each of three conditions: within a defended territory and a descendent of the locally dominant male (within-territory), within a defended territory but not a descendent of the locally dominant male (extra-territory), or in a non-defended territory where only non-dominant males were present (undefended); data summarized in Table 2. Consistent with treatment-level measurements, there were significantly more offspring born within-territory than in extra-territory or undefended conditions (GLMM: *n* = 36; *F* = 10.4, *P* = 0.0005). Promiscuous line males had higher overall reproductive success than monogamous line males across all three territory types (GLMM: *n* = 36; *F* = 8.13, *P* = 0.0083) (Figure 4C). There was no territory type *x* paternal treatment interaction (GLMM: *n* = 36; *F* = 0.3, *P* = 0.74), and post-hoc Tukey’s HSD tests showed no significant difference within any of the three conditions, indicating that greater reproductive success in promiscuous line males was driven by a moderate advantage in all three conditions.

#### Survivorship

**Figure 5:**
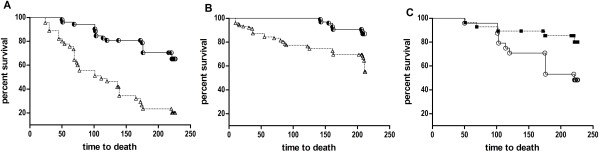
**Effects of social dominance and breeding treatment on survivorship. **Data are from 10 competition enclosures over 35 weeks. Regardless of treatment, socially dominant (half-filled circles) individuals had greater survivorship than subordinates (triangles) for both male (**A**) and female (**B**) founders. Promiscuous line males that were socially dominant (circles) had significantly lower survivorship than monogamous line males that were socially dominant (squares) (**C**).

There were no overall treatment effects on survivorship within males (*χ*^2^ = 2.32, *P* = 0.13) or females (*χ*^2^ = 0.15, *P* = 0.70). However, socially dominant males and females had greater survivorship relative to subordinates irrespective of treatment (males *χ*^2^ = 26.7, *P* = <0.0001; females *χ*^2^ = 15.1, *P* = 0.0001) (Figure 5A,B). A comparison of treatment effects of mortality among socially dominant males revealed significantly greater mortality in promiscuous line males relative to monogamous line males (Figure 5C; *χ*^2^ = 5.11, *P* = 0.024). There were no treatment effects on survivorship among socially dominant females (*χ*^2^ = 0.26, *P* = 0.61).

### Female mate choice: odor and mating preferences for promiscuous line males

The number of scent marks made by promiscuous line males (182.1 ± 40.4) was greater than that of monogamous line males (99.3 ± 29.3), and promiscuous line males also covered a greater area with scent marks (401.6 mm^2^ ± 91.4) than monogamous line males (255.8 mm^2^ ± 53.8), though the differences were not significant (GLM: number of marks, *F* = 2.11, *P* = 0.16; total area covered, *F* = 1.89, *P* = 0.19). Scent mark number or area covered did not influence the time of female investigation (GLM: number *F* = 0.054, *P* = 0.82; area *F* = 0.047, *P* = 0.83).

**Figure 6:**
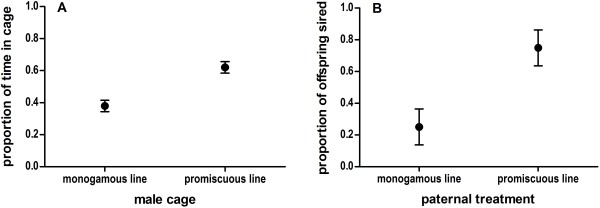
**Female preference for promiscuous line males vs. monogamous line males. **In a mate choice arena, females spent significantly more time in cages scent-marked by third-generation promiscuous line males (**A**), and had significantly more offspring sired by promiscuous line males (**B**), relative to monogamous line males. Transformed proportions were used in the analysis; raw proportions are shown for visual purposes only.

Females spent a greater proportion of time in cages scent-marked by promiscuous line males than monogamous line males (Figure 6A; t(10) = -3.33, *P* = 0.010). The same females also had a greater portion of offspring sired by promiscuous line males (Figure 6B; t(10) = -2.51, *P* = 0.036). As is typical of mouse mate choice experiments, we found frequent mating bouts (50.66 ± 12.28 per trial) during estrous [50], but due to a slow recording rate we were not able to distinguish intromissions from ejaculations. There were no significant effects of male breeding treatment on the frequency of the observed mating bouts (t(10) = -0.16, *P* = 0.88)). Five out of the nine litters conceived were pure litters of single parentage, all of which had promiscuous line paternity (binomial sign test: *P* = 0.002). In the three litters of mixed parentage, there were no treatment effects on male reproductive success (t(4) = 0.46, *P* = 0.68).

### Weight analysis: dynamic effects before and after sexual maturity

**Figure 7:**
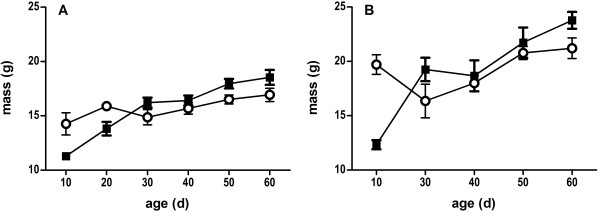
**Effect of breeding treatment on body mass before and after weaning. **Promiscuous line animals (circles) were heavier before weaning, but lighter after weaning, than monogamous line animals (squares). Means and standard errors are from an average of 10 caged mice each for females (**A**) and males (**B**).

The effect of breeding treatment on weight in male and female offspring is shown in Figure 7, and results from GLM analyses are summarized in Table 3. There were significant effects of treatment, age, and their interaction. Promiscuous line males and females were on average lighter than monogamous line animals from 30 to 60 days after birth. However, post-hoc Tukey’s HSD tests showed that promiscuous line males and females were significantly *heavier* at 10 days after birth. Post-hoc comparisons at other individual time points were not significant.

**Table 3:** Effects of breeding treatment on weight

**Source**	**Females**			**Males**		
	**SE**	**t**	***P***	**SE**	**t**	***P***
Treatment	0.17	1.95	0.053	0.32	1.94	**0.054**
Age	0.010	10.0	**<0.0001**	0.0072	10.8	**0.018**
Treatment × age	0.011	2.36	**0.020**	0.0072	2.40	**<0.0001**

GLM analysis with mass (g) as a dependent variable, and breeding treatment and age as fixed factors.

## Discussion

Following just two generations of reintroducing a wild-derived colony of laboratory mice to the process of social selection, we identified substantial, sex-specific lifetime fitness effects. The primary finding of this research is that, during direct competition among second-generation animals, promiscuous line males had greater mating success, but not greater viability, than monogamous line males; promiscuous line females had fewer litters than monogamous line females, and a significant treatment by sex interaction effect on reproductive success suggested a mild fitness load. Second, a mate choice experiment among third-generation animals showed that, when given a choice, females had odor and mating preferences for promiscuous line males over monogamous line males. Third, promiscuous line mice were significantly heavier than monogamous line mice 10 days after birth, but became lighter than monogamous line mice after weaning. Finally, although we did not experimentally test for genetic or nongenetic inheritance, transgenerational effects might have contributed to these results. We discuss each of these differences in turn.

During competition promiscuous line males had greater mating success (Figure 2A), but also reduced survivorship (Figure 5C), relative to monogamous line males, suggesting a functional trade-off between the expression of sexual traits and viability. Surprisingly, the promiscuous line male advantage could not be explained by greater success in acquiring social territories, a trait with a high heritability and a primary determinant of reproductive success [51]. Nor could it be explained by greater success in competitive ability during sperm competition, also a trait with high heritability [52] and an important component of reproductive success during social competition [8,9], because promiscuous line males had no advantage in polyandrous litters (Figure 4A). Rather, analysis of treatment-level reproductive success showed that promiscuous line males had nearly exclusive (95%) paternity within their territories, while monogamous line males had only 68% of the offspring born within their territories. Promiscuous line males also had higher paternity in undefended territories (Figures 3 and 4C). Parentage analysis confirmed that individual socially dominant promiscuous line males produced more “within-territory” offspring than dominant monogamous line males, and further revealed that they had greater success in mating outside their own territory (i.e., “extra-territory;” Figure 4C). Finally, promiscuous line males sired a greater number of litters, but not larger litters (Table 1).

In wild mouse societies, mate choice is likely driven by estrus females, who move between the territories of socially dominant males to sample prospective mates; dominant males usually don’t breed outside of their territories, and are not known to coerce females [33,35,36]. Although under studied, male preferences also exist in mice, and males who mate with their preferred partners benefit by producing offspring with increased fitness [6]. Our results suggest that female mating preference for an attractive “live fast die young” male phenotype largely drove the promiscuous line male advantage, as females that settled within territories defended by promiscuous line males were less likely to engage in extra-territorial mating than females settled in monogamous line male territories. We confirmed a role of female preference with a mate choice experiment on third-generation animals: when given a choice, females had both odor and mating preferences for promiscuous line males (Figure 6). Male mate choice might have also contributed to this effect. During direct competition, for instance, promiscuous line males may have refused to mate with low quality females or females that would not produce attractive male offspring. Alternatively, monogamous line males might have refused to mate with females that resided outside of their territorial boundaries. Because we did not explore the role of male choice in this experiment, we cannot rule out these possibilities.

Female fitness was relatively unaffected by breeding treatment during direct competition; there were no overall effects on reproductive success, survivorship or social dominance. Intriguingly, we found that promiscuous line females had significantly fewer litters than monogamous line females (though they had slightly but nonsignificantly more pups per litter). Consistently, our analysis demonstrated a significant treatment by sex interaction effect, where lower reproductive success in promiscuous line females was statistically correlated to higher reproductive success in promiscuous line males (Figure 2C). These sex specific results are consistent with the sexual conflict model, and support results from experiments in flour beetles and amphipods showing social selection pressures tend to enhance fitness in sons and deplete it in daughters [16,20]. However, our results contrast with studies on *D. melanogaster*, which often show strong negative effects of social competition [17-19].

Our results corroborate several studies in rodents that found fitness benefits of specific components of sexual selection (e.g., mate choice or sperm competition) by competing the offspring of experimental and control breeding treatments in seminatural arenas [5,6,8]. A study by Firman [9] bears particularly strong resemblance to ours. She found that, after 16 generations, male offspring from an enforced polyandry lineage had greater reproductive success than those from an enforced monogamous lineage when in direct competition in seminatual arenas; however, a behavioral mechanism for this advantage was not determined. Our work adds to these studies by showing that when all components of sexual selection are allowed to operate (without intervention) during competition for mating resources, there is an immediate, adaptive effect on male fitness.

“Live fast die young” and “sexy son” male phenotypes are classic signatures of sexual selection [53]. Two evolutionary genetic models of animal behavior could explain the male phenotype we observed. First, the sexy sons hypothesis predicts that a male who delivers no direct benefits (e.g., quality nesting sites) to the female can nevertheless be favored if his sons are sexually attractive. Alternatively, Zahavi’s handicap principal [26] predicts that sexually selected traits are honest indicators of health and vigor because they handicap performance and survival. Here, sexually selected traits provide a means for females to identify “good-genes” signals of quality in their mates because expression of the trait is negatively correlated with performance. Results from our experiment are perhaps more consistent with the sexy sons model, because promiscuous line males had an advantage in mating success and in attracting females, but were not of higher quality than monogamous line males. Furthermore, females of the promiscuous line were not of higher quality than females from the monogamous line. Importantly, these hypotheses are controversial because mathematical models [54] and experiments [17] suggest indirect effects via offspring fitness have trivial evolutionary effects relative to direct costs and benefits of female mating; our results add to growing evidence that indirect fitness effects can be substantial [55,56].

Although single-generation effects of sexual selection are often interpreted in light of genetic evolution [15], there is growing appreciation that the parental environment *per se* can significantly impact offspring phenotype and fitness independent of genetic inheritance, and that parental effects can ultimately influence the evolutionary response to sexual selection [41]. This is especially true for the social environment [57], and transgenerational effects have recently been reported in breeding experiments similar to ours [58]. Maternal effects on offspring fitness can arise by changes in the uterine hormonal milieu or nutritional investment in offspring, and effects on offspring weight are particularly common [41]. We found evidence for such effects in our weight analysis of F_3_ mice. Promiscuous line males and females were significantly heavier than monogamous line offspring before weaning, but were lighter after weaning (Figure 7). Because birth weight usually predicts adult weight in mammals, this shift was unexpected. During our study pregnant promiscuous line females were transferred from seminatural enclosures to solitary cages to give birth, and would be expected to have different stress hormone profiles than monogamous line females [59]. These differences could induce differential investment in offspring. Consistently, maternal stress late in pregnancy has previously been associated with elevated birth weight, and this weight difference diminished by postnatal week ten [60].

We are not able to determine if the advantage to promiscuous line males is due to genetic or transgenerational inheritance. Genetic selection could have occurred in the promiscuous line if particular alleles were strongly favored during social competition and sexual selection. Although MHC mating preferences have been observed in seminatural enclosures similar to ours [36], we eliminated any such potential effects by equalizing the frequency of MHC haplotypes between the two treatments. Also, although social dominance has been found to have a high narrow-sense heritability [61], we observed no selection on social dominance ability in our experiment. Finally, from our parentage analysis we found no significant differences in microsatellite heterozygosity between promiscuous line and monogamous line animals (data not shown). Nevertheless, other unidentified loci could have been under strong selection in this experiment. A non-mutually exclusive hypothesis is that transgenerational inheritance via maternal or paternal effects [62] could have increased mating success in promiscuous line males. We are currently investigating the role of transgenerational inheritance in this system.

## Conclusion

Individual components of sexual selection such as mate choice or intersexual competition can have dramatic effects on offspring development and fitness in the laboratory, but less is known about the total effects of sexual selection during naturalistic social competition. Although invertebrate studies that experimentally eliminate sexual selection using enforced monogamy show that broad-sense sexual selection has strong and deleterious effects on offspring fitness, these studies lack a critical feature of normal breeding systems: competition for mating resources. Social selection is an extended form of sexual selection that incorporates competitive social interactions and predicts complex evolutionary dynamics [12,13].

We report here new results on the effects of social selection on vertebrate offspring fitness, and show that sons conceived during social competition have a fitness advantage by having greater reproductive success and attractiveness to females. This latter effect involves chemical communication and, likely, the expression of pheromones. We also identify fitness tradeoffs, in the form of male survivorship and female fecundity, which accompany adaptation to social selection. These data suggest that sexually selected phenotypes are lost during standard laboratory breeding procedures, but are quickly regained when all components of sexual selection are allowed to operate during social competition for mating resources.

## Competing interests

The authors declare that they have no competing interests.

## Authors’ contributions

ACN and WKP conceived of the study. ACN carried out the experiments and drafted the manuscript. KEC and SH participated in the direct competition and mate choice experiments. WKP participated in coordination of the study. All authors read and approved the final manuscript.
